# Retraction Note: Emerging role of mesenchymal stem/stromal cells (MSCs) and MSCs-derived exosomes in bone- and joint-associated musculoskeletal disorders: a new frontier

**DOI:** 10.1186/s40001-025-03274-z

**Published:** 2025-10-04

**Authors:** Mohammad Hadi Gerami, Roya Khorram, Soheil Rasoolzadegan, Saeid Mardpour, Pooria Nakhaei, Soheyla Hashemi, Bashar Zuhair Talib Al-Naqeeb, Amir Aminian, Sahar Samimi

**Affiliations:** 1 Bone and Joint Diseases Research Center, Shiraz University of Medical Sciences, Shiraz, Iran; 2 Bone and Joint Diseases Research Center, Department of Orthopedic Surgery, Shiraz University of Medical Sciences, Shiraz, Iran; 3 Department of Surgery, School of Medicine, Shahid Beheshti University of Medical Sciences, Tehran, Iran; 4 Department of Radiology, Imam Khomeini Hospital, Tehran University of Medical Sciences, Tehran, Iran; 5 Endocrinology and Metabolism Research Center (EMRC), Vali-Asr Hospital, Tehran University of Medical Sciences, Tehran, Iran; 6 Obstetrician, Gynaecology & Infertility Department, Isfahan University of Medical Sciences, Isfahan, Iran; 7 Anesthesia Technology Department, Al-Turath University College, Al Mansour, Baghdad, Iraq; 8 Bone and Joint Reconstruction Research Center, Shafa Orthopedic Hospital, Iran University of Medical Sciences, Tehran, Iran; 9 Tehran University of Medical Sciences, Tehran, Iran

**Retraction: European Journal of Medical Research (2023) 28:86** 10.1186/s40001-023-01034-5

The Editor-in-Chief has retracted this article. An investigation by the Publisher found evidence of authorship manipulation. The Editor-in-Chief therefore no longer has confidence in the reliability of the results and conclusions of this article.

Roya Khorram disagrees with this retraction. Sahar Samimi has not stated whether they agree or disagree with this retraction. The remaining authors have not replied to correspondence from the Publisher about this retraction.

